# Bacterial aggregation triggered by low-level antibiotic-mediated lysis

**DOI:** 10.1038/s41522-024-00553-1

**Published:** 2024-09-26

**Authors:** Sharareh Tavaddod, Angela Dawson, Rosalind J. Allen

**Affiliations:** 1 School of Physics and Astronomy, University of Edinburgh, Edinburgh, UK; 2 Theoretical Microbial Ecology, Institute of Microbiology, Faculty of Biological Sciences, Friedrich Schiller University Jena, Jena, Germany; 3 Cluster of Excellence Balance of the Microverse, Friedrich Schiller University Jena, Jena, Germany

**Keywords:** Biofilms, Antimicrobials

## Abstract

Suspended bacterial aggregates play a central role in ocean biogeochemistry, industrial processes and probably many clinical infections – yet the factors that trigger aggregation remain poorly understood, as does the relationship between suspended aggregates and surface-attached biofilms. Here we show that very low doses of cell-wall targeting antibiotic, far below the minimal inhibitory concentration, can trigger aggregation of *Escherichia coli* cells. This occurs when a few cells lyse, releasing extracellular DNA – thus, cell-to-cell variability in antibiotic response leads to population-level aggregation. Although lysis-triggered aggregation echoes known trigger mechanisms for surface-attached biofilms, these aggregates may have different ecological implications since they do not show increased biofilm-forming potential or increased antibiotic resistance. Our work contributes to understanding the nature of bacterial aggregates and the factors that trigger their formation, and the possible consequences of widespread low-dose antibiotic exposure in the environment and in the body.

## Introduction

Many microbes form aggregates in liquid suspension. Aggregation can alter microbial physiology, enhancing antibiotic tolerance and biofilm formation^1–5^. It can also have important clinical, biophysical, and ecological effects^2,6,7^, including aggregate sinking that drives sequestration of carbon in the ocean and in wastewater treatment plants, preferential expulsion from the gut^8^, and loss of ability to invade a host^9^. Microbial aggregates have even been proposed as an early form of multicellular life^10^. However, despite its ubiquity and importance, in most cases, the environmental factors that trigger aggregation, and the mechanisms by which they act, remain unclear.

Several previous reports have linked low-dose antibiotic treatment with aggregation in aquatic bacterial communities^11^ and for *i*n vivo gut bacteria in a zebrafish model^8^, although the mechanisms are unclear. These findings are potentially significant because microbial exposure to low-dose antibiotics is widespread – occurring in wastewater, rivers, and lakes, as well as during antibiotic therapy, agricultural use of antibiotics, or due to ecological interactions between microbes (e.g. in soil). Low-dose antibiotic exposure is already a cause of concern because of its potential to enrich for antibiotic-resistant mutants^12^ or to trigger quorum-sensing, virulence, and biofilm formation^12–15^. Here we develop an in vitro *Escherichia coli* model system for the aggregative effects of low-dose antibiotic exposure. In this model system, bacterial aggregation can be triggered by exposure to antibiotics at very low doses (many times lower than the minimal inhibitory concentration at the equivalent cell density).

It is well known that microbial aggregation can be mediated by diverse mechanisms, including exopolysaccharides, extracellular DNA (eDNA) or proteins, and chemotaxis^1,4,16–21^. Up to now the role of heterogeneity among individual cells in the population has not been much discussed, although a pioneering study showed that eDNA is generated in *P. aeruginosa* liquid cultures via lysis of a subpopulation of cells in a process that is dependent on quorum-sensing, flagella and type IV pili^18^. For antibiotic-triggered aggregation, our work suggests a central role for eDNA as the cohesive agent. We link the release of cohesive eDNA to the antibiotic-mediated lysis of a small subpopulation of bacteria. Since the vast majority of bacteria do not lyse at these very low antibiotic concentrations, it appears that the stochastic lysis of a few atypically antibiotic-sensitive cells within the population can drastically alter the fate of the population as a whole.

The similarities and differences between bacterial aggregates in liquid and surface-attached biofilms are a topic of increasing discussion^7^. Multiple studies have found that bacteria in aggregates have physiological characteristics similar to those found in biofilms, including reduced antibiotic susceptibility^1–4,22^. This has led to the idea that aggregates can be considered as ‘non-surface-attached biofilms’^1,7^, and/or as seeds for biofilm growth^5,23^. Moreover, biofilm formation can be triggered by the release of eDNA, caused by the lysis of a subpopulation of bacteria - triggered by antibiotics, detergent, prophages, or phage genes^13–15,20,24–29^. While the aggregation mechanism identified in our work echoes eDNA-triggered biofilm formation, we find no evidence for these aggregates acting as a precursor to biofilm formation - on the contrary, lysis-triggered aggregation appears to suppress biofilm formation. Moreover, while biofilms often show increased antibiotic resistance (the minimal concentration needed to inhibit biofilm growth is often much higher than that needed to inhibit planktonic growth^30^), we observe no change in the minimal inhibitory concentration caused by aggregation (although we do not rule out changes in antibiotic tolerance^1–4,22^).

Taken together, our work shows that low-dose antibiotic exposure can trigger the formation of *E. coli* aggregates in liquid suspension. For dense, well-shaken suspensions of *E. coli*, we find that very low concentrations of the *β*-lactam antibiotic mecillinam cause the formation of bacterial aggregates within hours. We link aggregation to the lysis of a small subpopulation of bacteria, releasing eDNA that mediates bacterial cohesion. Aggregation does not increase the minimal inhibitory concentration for antibiotic inhibition, nor does it enhance biofilm formation. Our work contributes to increasing understanding of the nature of bacterial aggregates and the factors that can trigger their formation, as well as of the potential consequences of widespread low-dose antibiotic exposure in the environment and in the body.

## Results

### Low-dose *β*-lactam antibiotics cause bacterial aggregation via lysis of a few cells

To investigate the effects of subinhibitory antibiotic exposure on liquid-phase bacterial cultures, we grew cultures of *E.* *coli* strain MG1655 in MOPS minimal media with glucose to an optical density OD_600_~0.2, corresponding to about 1.6 × 10^8^ cells/ml (see “Methods” section), in a shaken conical flask. We then added very low doses of antibiotic, corresponding to 1/8000 times the minimum inhibitory concentration (MIC) value, measured under the same conditions (MIC^OD0.2^; see “Methods” section and Table 1). The cultures were sampled taken over a period of 5 h after antibiotic addition and examined using phase-contrast microscopy. At such low concentrations, the antibiotic did not significantly affect the population-level growth dynamics, as measured either by spectrophotometry or by viable counts (Supplementary Figure S1).

**Table 1:** Minimum inhibitory concentration (MIC) for mecillinam (Mec.), aztrenoam (Azt.), streptomycin (Str.), tetracycline (Tet.), and rifampicilin (Rif.) for *E. coli* strain RJA002 in MOPSGlu medium at 37 °C

Antibiotic		Mec.	Azt.	Str.	Tet.	Rif.
MIC^OD0.2^	(μg/ml)	840	640	3 ± 1	1.5 ± 0.5	100 ± 20
MIC^OD0.8^	(μg/ml)	N.M.	N.M.	3 ± 1	2 ± 1	40 ± 10
MIC^Mec.^	(μg/ml)	N.M.	N.M.	2 ± 1	0.4 ± 0.1	20 ± 10
MIC^Mec./DNase^	(μg/ml)	N.M.	N.M.	2 ± 1	0.4 ± 0.1	20 ± 10

MIC values were measured for cultures under different conditions: cultures at OD 0.2 at the time of antibiotic addition (MIC^OD0.2^); cultures at OD 0.8 at the time of antibiotic addition (MIC^OD0.8^); cultures that had been pre-incubated with Mec. (at MIC^OD0.2^/8000) for 4 h to induce aggregate formation (MIC^Mec.^); cultures that pre-incubated with Mec. for 4 h then treated with DNase I (MIC^Mec./DNase^) to disperse the aggregates (see “Methods” section). For details of the MIC measurement protocol, see “Methods” section. N.M. indicates ‘not measured’. The ‘low-dose’ antibiotic concentrations used in this work and referred to in the text as MIC^OD0.2^/8000 were 1/8000 times the values reported in the top row of the table, i.e. 0.1 μg/ml for mecillinam, 0.08 μg/ml for aztreonam, 0.0004 *μ*g/ml for streptomycin, 0.0002 μg/ml for tetracycline and 0.01 μg/ml for rifampicin.

#### Mecillinam and aztreonam cause morphological changes and aggregation

**Fig. 1:**
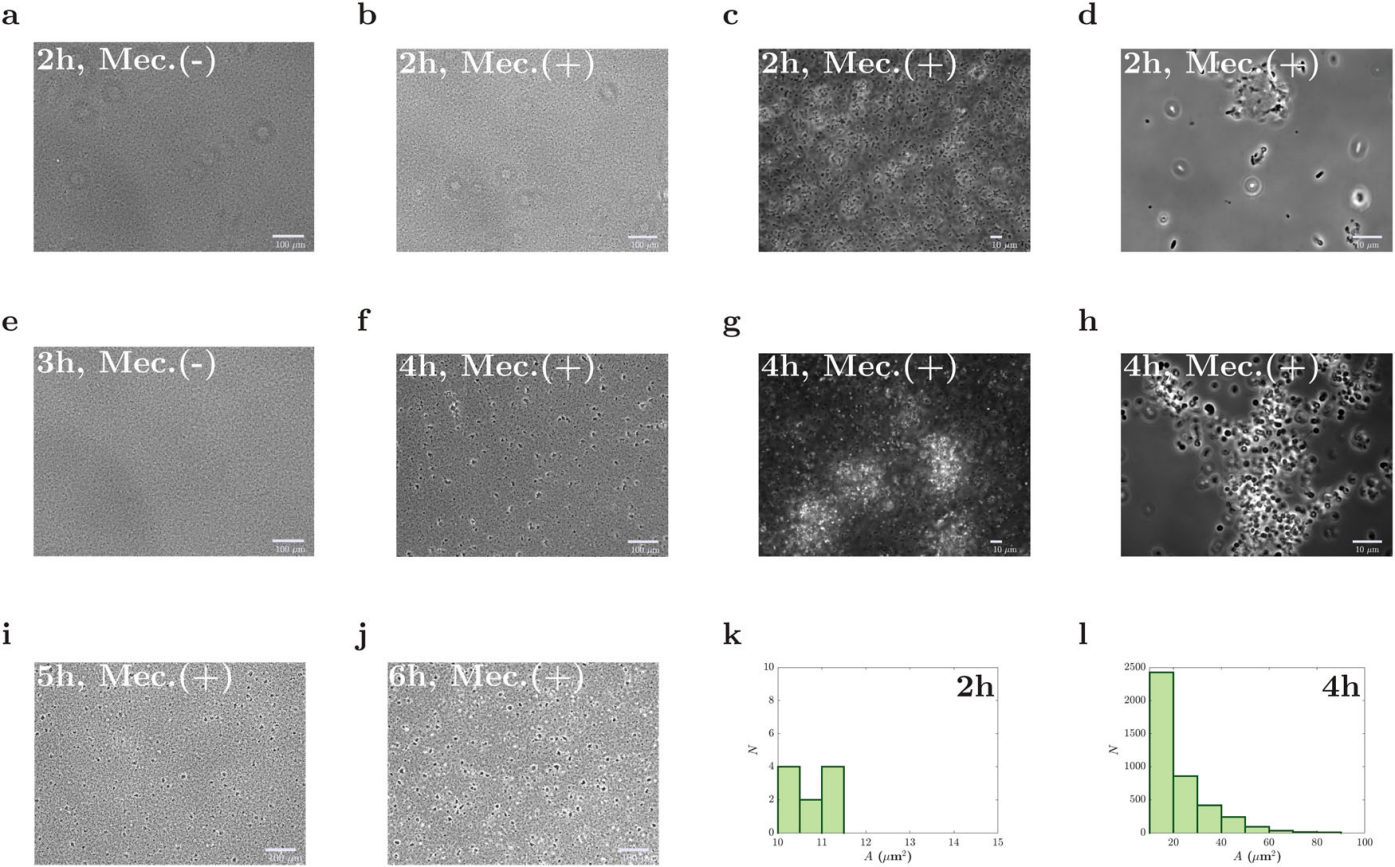
Aggregation upon incubation with mecillinam. Cultures of *E. coli* MG1655 at OD 0.2 were incubated without (control; Mec (−)) and with mecillinam (Mec (+)) at MIC^OD0.2^/8000 = 0.1 μg/ml, in shaken flasks (see Table 1). Phase-contrast images for cell culture samples taken at 2 h (top; **a**–**d**), 3 h (middle; **e**), 4 h (middle; **f**–**h**), 5 h (bottom; **i**), and 6 h (bottom; **j**) are shown at different magnifications (10× (**a**, **b**, **e**, **f**, **I**, **j**), 40× (**c**, **g**), and 100× (**d**, **h**)). **k**, **l** show the distribution of cluster size: the average number of clusters, *N* of area, *A* (larger than 10 μm^2^), is shown as a histogram. Data was obtained over 10 different fields of view, 2 h (**k**) and 4 h (**l**) after addition of mecillinam.

We tested four antibiotics - mecillinam, aztreonam, streptomycin, and tetracycline. Mecillinam and aztreonam are cell-wall synthesis-targeting *β*-lactam antibiotics, while streptomycin and tetracycline bind to ribosomes, inhibiting protein synthesis^31,32^. Both the *β*-lactam antibiotics caused morphological changes, lysis of a few cells, and the formation of cell aggregates over a period of 2–4 h (Fig. 1; Supplementary Fig. 2). However, for the two protein synthesis-targeting antibiotics we observed no change in cell morphology and no aggregate formation (Supplementary Fig. 3).

It is well known that *β*-lactam antibiotics cause morphological changes and lysis at high concentrations, close to or above the MIC. Mecillinam inhibits the synthesis of peptidoglycan by binding to the PBP2 transpeptidase enzyme that is required for cell elongation, causing cells to become round and lyse^33^, while aztreonam inhibits cell division by targeting the PBP3 transpeptidase, which is involved in the synthesis of cell pole peptidoglycan - hence it causes filamentation and eventually lysis^34^. Even though the concentrations of mecillinam or aztreonam in our experiments were far below the MIC, we observed similar morphological changes. For mecillinam, the cell shape changed from rod-shaped to round, and swimming motility stopped, about 2 h after the addition of the antibiotic. For aztreonam, we observed filamentation and loss of motility (Supplementary Fig. 2).

For both mecillinam and aztreonam, we observed the formation of aggregates consisting of 4–6 cells, about 2 h after the addition of antibiotic (Fig. 1; Supplementary Figs. S4 and S5). After a further 30 min, these aggregates had become larger, and they continued to grow over the next 4 h (Fig. 1). The aggregates appear to increase in abundance over time (Fig. 1): we did not observe aggregate dispersal at long times as has been reported for *Pseudomonas aeruginosa*^1,35^.

#### Low-dose mecillinam causes a very low rate of cell lysis

Motivated by reports of biofilm formation triggered by *β*-lactam mediated lysis^13–15^, we hypothesized that the very low levels of cell lysis that we observed via microscopy (Supplementary Fig. 2) might be relevant for aggregation - even though the population as a whole continues to grow under these very low levels of antibiotic (Supplementary Fig. 1). To quantify the rate of antibiotic-induced cell death in our cultures, we used resazurin, a weakly fluorescent, nontoxic, cell-permeable dye which, in the presence of metabolically active cells, is irreversibly transformed to the highly fluorescent resorufin^36^. We measured resorufin fluorescence emission for exponentially growing bacterial cultures in the presence or absence of low-dose mecillinam (see “Methods” section). Comparing the exponential rate of fluorescence increase for cultures with and without mecillinam, we concluded that about 6.8% of cell lifetimes end in lysis rather than in further division (see “Methods” section). This low rate of cell lysis is not detectable when we compare growth curves measured via either optical density or colony-forming units in the presence and absence of mecillinam (Supplementary Fig. 1; see “Methods” section).

### Aggregate formation is mediated by extracellular DNA

Extracellular DNA (eDNA) released from lysed cells has been found to play an important role in antibiotic-triggered biofilm formation^13–15^. Therefore we speculated that released components of the lysed cells in our cultures, such as DNA or proteins, might mediate aggregation of the remaining, non-lysed cells.

#### eDNA is present in aggregates and appears to bridge between cells

**Fig. 2:**
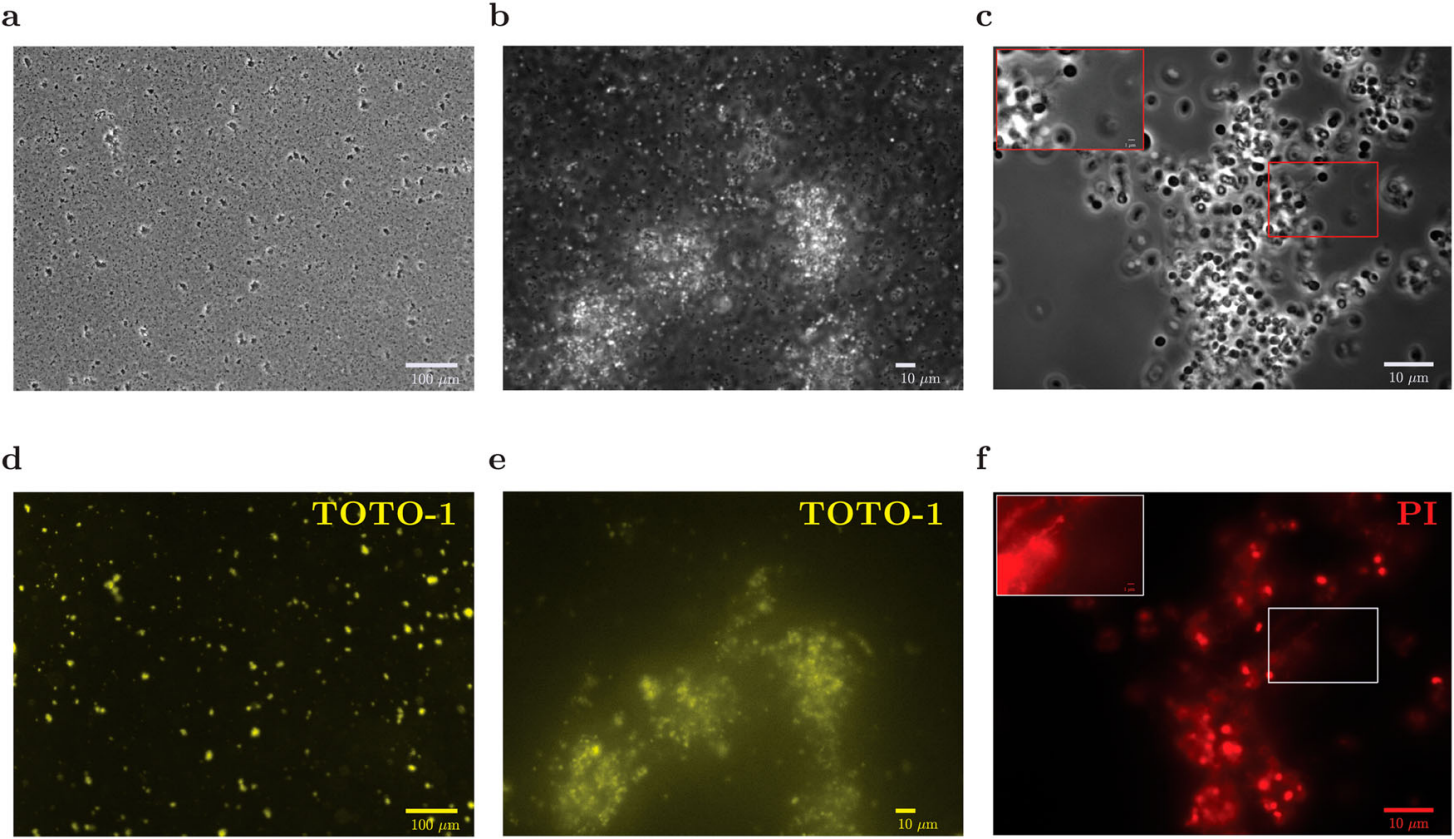
Aggregates contain eDNA. Cultures of *E*. *coli* MG1655 at OD 0.2 were incubated with mecillinam at MIC^OD0.2^/8000 = 0.1 μg/ml, in shaken flasks (see Table 1). Samples were taken 4 h after the addition of mecillinam and stained with either TOTO-1, which binds to eDNA, or propidium iodide (PI), which binds to both eDNA and the DNA of membrane-damaged cells. The top row shows the same phase-contrast images as in Fig. 1**f**–**h**, with magnification increasing left to right: **a** 10×, **b** 40×, and **c** 100×. The bottom row shows the corresponding fluorescence images for TOTO-1 (**d**, **e**) and PI (**f**). In (**c**) and (**f**), the upper left inset shows an expanded image of the central boxed region in which the intensity of each pixel has been increased by a factor of 3.8. The inset shows the presence of eDNA bridges between cells.

To investigate the role of eDNA in the structure of the aggregates, we first performed microscopic imaging in the presence of several DNA-binding dyes. TOTO-1 is often used to stain eDNA, since it increases its fluorescence intensity by 100- to 1000-fold upon binding large fragments of DNA (10–50 kbp.)^37–39^. TOTO-1 binds to both dsDNA and ssDNA with similar affinity^40^; it has low penetrance of the cell membrane and therefore is not expected to stain chromosomal DNA. Propidium iodide (PI) has similar properties, but it has higher membrane penetrance and is therefore expected to stain both eDNA and chromosomal DNA of cells with damaged membranes^41^. We added TOTO-1 or PI to dense planktonic cultures, adding at the same time low-dose mecillinam or aztreonam to promote aggregation. We then imaged the cultures after 4 h. Both dyes revealed the presence of eDNA in the aggregates (Fig. 2; see also Supplementary Figs. S4 and S5). For both dyes, fluorescence was most intense at the positions where aggregates were observed by phase-contrast microscopy (Fig. 2d, e for TOTO-1; Fig. 2f for PI). We were even able to observe apparent eDNA ‘bridges’ between cells within the same aggregate (upper left inset of Fig. 2f).

#### Digestion of eDNA, but not eProtein, eliminates aggregation

**Fig. 3:**
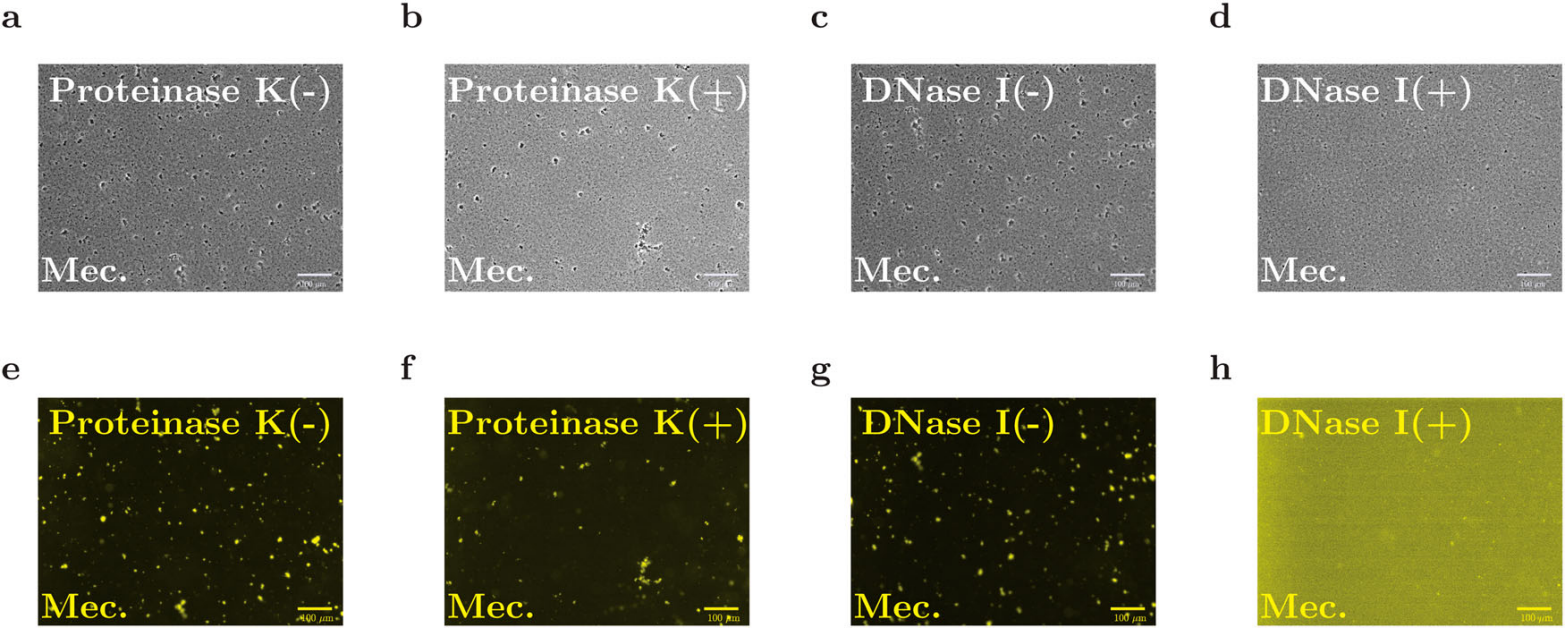
DNAse I treatment disperses pre-formed aggregates, while the addition of proteinase K does not destroy aggregates. Phase-contrast images and the corresponding TOTO-1 fluorescence images, for the wild-type MG1665 strain, incubated with mecillinam at MIC^OD0.2^/8000, in shaken flasks (see Table 1), for 4 h, in the presence and absence of Proteinase K (**a**, **b**, **e**, **f**) and DNase I (**c**, **d**, **g**, **h**). Proteinase K (20% *v*/*v*) or DNAse I (10% *v*/*v*) were added at the end of the experiment (see “Methods” section). Scale-bar = 100 μm.

To probe the possible roles of eDNA and extracellular protein (eProtein) in aggregation, we generated cultures containing aggregates by incubating with low-dose mecillinam for 5 h (see “Methods” section), then added either deoxyribonuclease I (DNase I), to digest eDNA, or proteinase K^2,42^, to digest eProtein (see “Methods” section). After adding DNase I, the aggregates quickly disappeared and the TOTO-1 signal became homogeneous across the sample (Fig. 3; see also Supplementary Fig. 4). In contrast, addition of proteinase K did not destroy the aggregates, even when we increased the concentration and incubation time (Fig. 3); see also Supplementary Fig. 6. We verified that the aggregate size distribution remained the same with or without addition of proteinase K, even though the addition of the proteinase K somewhat diluted the sample (Supplementary Fig. 6).

Equivalent results were obtained upon adding DNase I or proteinase K to aggregates formed by incubating with low-dose aztreonam: again, pre-existing aggregates were destroyed by digestion of eDNA but not by digestion of eProtein (Supplementary Figs. S5 and S6).

**Fig. 4:**
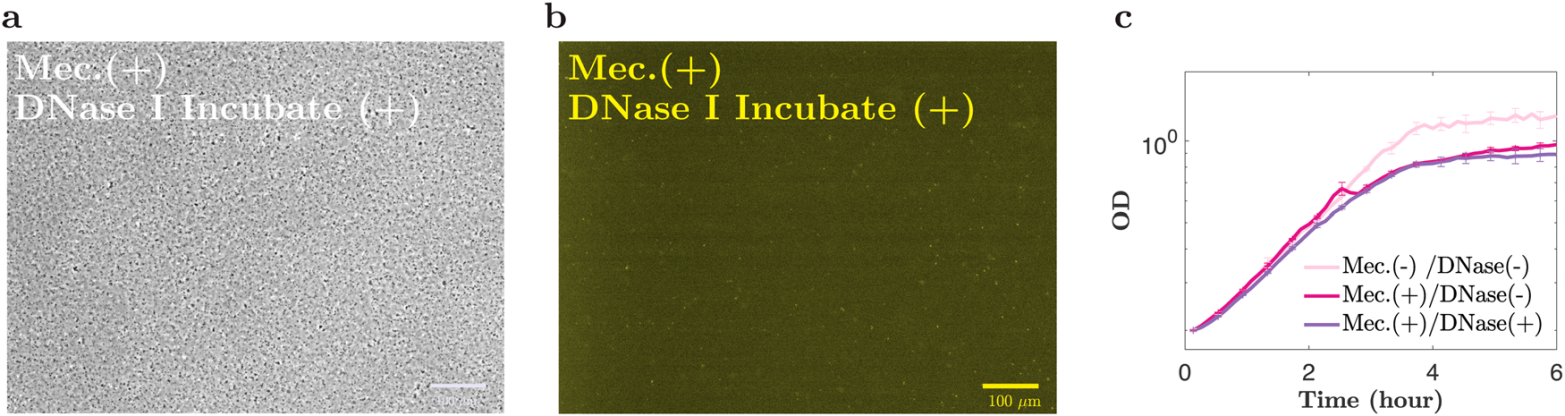
Incubation with DNAse I prevents aggregation. **a**, **b** Phase-contrast image and the corresponding TOTO-1 fluorescence image, for the wild-type MG1665 strain, incubated with mecillinam at MIC^OD0.2^/8000, in a shaken flask (see Table 1), in the presence of DNase I (5% *v*/*v*, from the start of the experiment), for 4 h. **c**: Growth curve (OD vs time) for the same cell culture, with and without mecillinam, and/or DNase I. Scale-bar = 100 μm.

We also observed that DNase I could prevent aggregates from forming in the first place. Repeating our experiment, but this time adding DNase I to the dense planktonic culture at the start, together with low-dose mecillinam (or aztreonam), we observed no aggregate formation over a 5-h period (Fig. 4 for mecillinam; Supplementary Fig. 7 for aztreonam). In contrast, when we added proteinase K at the start of the experiment together with low-dose mecillinam, aggregates formed, suggesting that eProtein is not involved in the aggregation process (Supplementary Fig. 8).

Repeating the same experiment with aztreonam plus proteinase K, we did not observe aggregate formation (Supplementary Fig. 8), but it appeared that proteinase K might have inhibited the action of aztreonam since we observed no change in the shape or swimming motility of the bacteria in this experiment (in contrast to our other experiments with aztreonam).

These results suggest that aggregation in our experiments is mediated by eDNA, which is released when a minority of cells in the culture lyse under the action of the cell-wall targeting antibiotic.

#### Spectrophotometry supports that eDNA release mediates aggregation

**Fig. 5:**
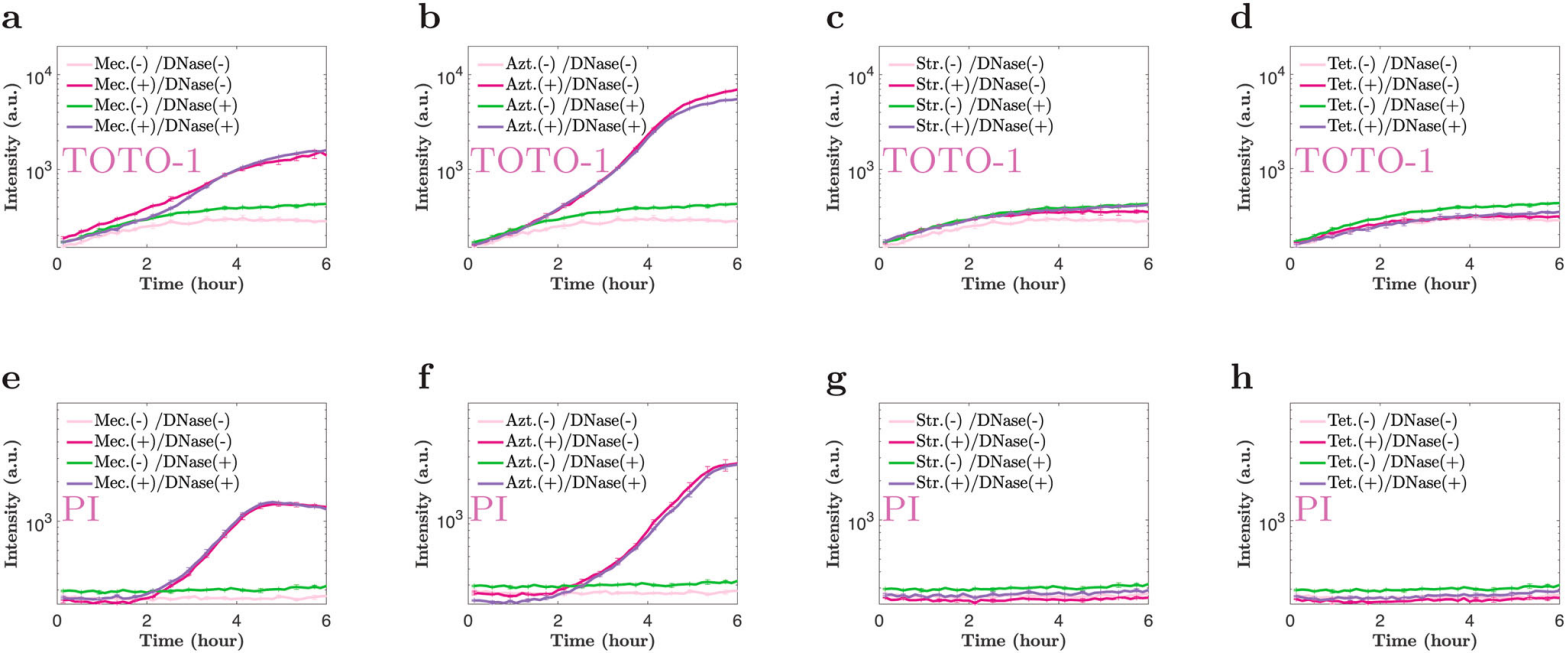
eDNA is released upon incubation with low-dose mecillinam or aztreonam, but not upon incubation with low-dose streptomycin or tetracycline. Cultures of *E. coli* MG1655 at OD 0.2 were incubated with mecillinam, aztreonam, streptomycin, or tetracycline at MIC^OD0.2^/8000 (see Table 1) on MOPSGlu media at 37 °C, in shaken flasks, together with either TOTO-1, which binds to eDNA or propidium iodide (PI), which binds to both eDNA and the DNA of membrane-damaged-cells. TOTO-1 and PI emission were measured using bulk spectrophotometry as a function of time (after the addition of antibiotic), for cultures with and without antibiotic, and with and without DNase I. **a**–**d** show results for mecillinam, aztreonam, streptomycin or tetracycline and TOTO-1. **e**–**h** show results for mecillinam, aztreonam, streptomycin, or tetracycline and PI. The cultures incubated with mecillinam and aztreonam show increased fluorescence corresponding to eDNA release but the cultures incubated with streptomycin or tetracycline do not. The data are expressed as mean ± standard error of the mean, for four replicate experiments. Scale-bar = 10 μm.

Since both TOTO-1 and PI increase their fluorescence intensity upon binding to DNA, we can also use bulk spectrophotometry measurements to detect changes in the amount of eDNA in the culture. Figure 5a, b shows time series data for TOTO-1 emission intensity over 6 h, following the addition of low-dose mecillinam (a) or aztreonam (b). The intensity of TOTO-1 emission increased by about 10-fold, 1–2 h after the addition of the cell-wall targeting antibiotic, consistent with the time at which cells were observed to change morphology and begin to aggregate. Similar results were obtained for the PI emission intensity (Fig. 5e, f), supporting the view that eDNA release mediates aggregation. The release of DNA was observed in these experiments even in the presence of DNAse I (Fig. 5a, b, e, f), consistent with DNase I being able to act on the eDNA only after it is released. Since the dynamics of fluorescence emission were largely unaffected by the presence of DNase I, we speculate that the binding of the dye to the eDNA might interfere with DNase I activity.

Repeating these experiments using the ribosome-targeting antibiotics streptomycin and tetracycline, for which we did not observe aggregation (Supplementary Fig. 3), we observed no significant increase in emission intensity for either TOTO-1 or PI (Fig. 5c, d, g, h), supporting our hypothesis that lysis caused by cell-wall targeting antibiotics was responsible for the eDNA release.

#### Purified genomic DNA does not trigger aggregation

If aggregation is mediated by eDNA, one might expect that the addition of purified genomic DNA (gDNA) would cause cells to aggregate, even in the absence of cell-wall targeting antibiotics. However, we did not observe aggregation when we added gDNA (from *E. coli* strain B) to a dense *E*. *coli* culture (OD_600_ = 0.2; strain MG1655). The concentration of gDNA added (0.25–20 μg/ml) was equivalent to the concentration of eDNA estimated to be released by cell lysis in our mecillinam cultures (see “Methods” section). We repeated these experiments using *E. coli* strain AD37, which is unable to swim due to paralyzed flagella (see “Methods” section), to mimic the cessation of swimming motility that happens upon addition of low-dose mecillinam or aztreonam (having verified that strain AD37 forms aggregates upon addition of low-dose mecillinam and aztreonam - Supplementary Fig. 9). We also varied the optical density of the AD37 cultures, testing cultures at OD_600_ = 0.2 and 0.4. However, no aggregation was observed.

This result is reminiscent of the observation of Turnbull *e*t al. that the exogenous addition of *P. aeruginosa* gDNA did not restore biofilm formation in a mutant that was unable to release eDNA via prophage-mediated “explosive cell lysis”^29^. Turnbull et al. found that contrary to expectations, exogenous gDNA actually inhibited microcolony formation^29^. They speculated that eDNA might need to be provided in high local concentrations to trigger biofilm initiation, or that other components might be required in addition to eDNA. Our results may support the suggestion that other factors are required to trigger, or mediate, aggregation, in addition to eDNA. However, we note also that the eDNA that is released in our experiments upon antibiotic lysis is likely to differ from purified gDNA, since DNA purification degrades DNA-associated proteins, decreases the viscosity of the DNA, and may alter the composition of counter-ions on the DNA^42–44^.

### Ecological consequences of aggregate formation

Previous work has linked bacterial aggregation with decreased susceptibility to antibiotics^2–4^, biofilm-like bacterial physiology^1^, and the seeding of surface-attached biofilms^5^, leading to speculation that bacteria in aggregates might be in a biofilm-like physiological state, or that bacterial aggregates could be considered as a ‘third lifestyle’^7^. Therefore we wondered whether the bacterial aggregates that are triggered in our system by low doses of cell-wall targeting antibiotics might show altered susceptibility to other antibiotics (at much higher doses), and/or increased propensity for biofilm formation.

#### Aggregation does not increase the minimal inhibitory concentration for antibiotic inhibition

For bacteria in biofilms, the minimal concentration of antibiotic that is required to inhibit growth is often much higher than for planktonic bacteria^30^. To determine whether aggregate formation in our system altered the minimal inhibitory concentration for antibiotic inhibition, we measured MIC values (MIC^Mec.^) for the antibiotics tetracycline, streptomycin, and rifampicin, four hours after the addition of low-dose mecillinam to a dense culture to induce aggregation (see “Methods” section and Table 1). Tetracycline and streptomycin had previously been found not to cause aggregation (Fig. 5, Supplementary Fig. 3). For comparison, we performed two control experiments. In the ‘DNase-dispersed’ control experiment, aggregates were dispersed by adding DNase I just before the MIC measurement (MIC^Mec./DNase^). In the ‘no-mecillinam’ control experiment we measured MIC values for equivalent cultures that had been incubated for 4 h without the addition of mecillinam, reaching an OD of ~0.8 (MIC^OD0.8^). If aggregation makes bacteria more resistant to antibiotics, then we would expect to obtain higher MIC values for the aggregated samples (MIC^Mec.^) compared to either the dispersed control (MIC^Mec./DNase^) or the no-mecillinam control (MIC^OD0.8^). Comparing the MIC measurements for the aggregated samples (MIC^Mec.^) with the DNase-dispersed control (MIC^Mec./DNase^) we observed no significant difference (Table 1). Therefore the presence of aggregates, in itself, has no effect on resistance to antibiotics in our system.

However, a more complex picture emerged upon comparing the MIC values for the no-antibiotic control (MIC^OD0.8^) with those for the aggregated samples (MIC^Mec.^) or the DNase-dispersed control (MIC^Mec./DNase^). For tetracycline, MIC^OD0.8^ was significantly higher than MIC^Mec.^ or MIC^Mec./DNase^. This suggests that very low-dose mecillinam treatment can increase the resistance of *E. coli* to tetracycline (by a factor of 5), in a manner that is independent of aggregate formation. For streptomycin and rifampicin, the apparent difference between MIC^Mec./DNase^, MIC^Mec.^ and MIC^Mec./DNase^ was not significant. Previous studies of the combined effect of tetracycline and *β*-lactam antibiotics (at much higher concentrations) suggest that these antibiotic classes tend to be antagonistic for *E. coli* cultures at low density on rich medium: i.e. the drugs are less effective when combined than they would be individually^45,46^. Therefore our result that low-dose mecillinam can potentiate the action of tetracycline hints that environmental conditions (imbalance in concentration between antibiotics, growth medium, and cell density) might alter the nature of drug interactions.

Biofilm formation can enhance not only antibiotic resistance (i.e. minimal inhibitory concentration) but also antibiotic tolerance (the ability of bacterial to survive antibiotic treatment of a given time duration)^47^. Several previous studies have reported increased antibiotic tolerance for bacteria in aggregates^1–4,22^. Since our measurements focused on MIC values rather than killing dynamics, we cannot rule out that aggregation might also alter antibiotic tolerance in our system.

#### Biofilm formation is stimulated by low-dose antibiotics but impeded by aggregation

Low-dose antibiotic treatment has previously been found to enhance the formation of biofilms on surfaces^13–15^, while other studies have suggested that cell aggregates may be a precursor to biofilm formation^5,7,23^. To determine whether biofilm formation was affected by low-dose mecillinam treatment and/or the resulting cell aggregation, we performed microtitre plate biofilm assays. Briefly, cultures were incubated under static conditions in a 96-well microplate for 15 h, then biofilm formation was assayed using safranin-O staining (see “Methods” section and Supplementary Fig. 10). To disentangle the effects of aggregation and of mecillinam treatment, we compared biofilm formation for aggregated cultures (5 h after addition of low-dose mecillinam), no-mecillinam control cultures (which had been incubated for 5 h without the addition of mecillinam), DNase-dispersed control cultures (which had been incubated with mecillinam for 5 h then treated with DNase I to disperse aggregates) and vortex-dispersed control cultures (in which aggregates had been dispersed by intense mixing).

**Fig. 6:**
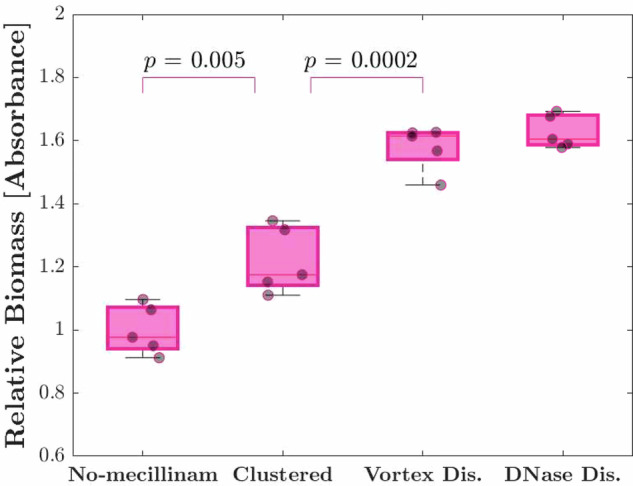
Low-dose mecillinam treatment enhances biofilm formation but aggregation does not. Relative biofilm formation was quantified in a microplate assay for (i) no-mecillinam control cultures (MG1655, MOPSGlu medium, 37 °C, initial OD 0.2, which had been incubated for 5 h without addition of mecillinam); (ii) aggregated cultures (as in (i) but incubated for 5 h with low-dose mecillinam at MIC^OD0.2^/8000); (iii) vortex-dispersed (Vortex Dis.) control cultures (as in (ii) but aggregates were mechanically disrupted); and (iv) DNase-dispersed (DNase Dis.) control cultures (as in (ii) but aggregates were chemically disrupted using DNase I) -- see “Methods” section for details. Biofilms were allowed to form for 15 h in 96-well microplates under static conditions, washed, and stained with safranin-O. Absorbance was measured at 510 nm and normalized to the mean absorbance of the no-mecillinam control samples. Each data point represents a single biological replicate (mean of 20–24 technical replicates). The bottom and top edges of each box indicate the 25th and 75th percentiles of five biological replicates and the red line in each box represents the median of five biological replicates. Error bars in each box are the 95% confidence interval of five biological replicates.

We observed a significant increase in biofilm formation for all the cultures that had been treated with low-dose mecillinam – i.e. the aggregated cultures and the two cultures in which aggregates had been dispersed (Fig. 6). Therefore, low-dose mecillinam treatment led to increased biofilm formation, consistent with previous work with other cell-wall targeting antibiotics and bacterial species^14,15^. However, this increase in biofilm formation was not mediated by aggregation. Comparing our results for the aggregated cultures to the DNase-dispersed and vortex-dispersed control cultures, we found that the dispersed control cultures actually formed significantly more biofilm than the aggregated cultures (Fig. 6). Therefore, aggregated cells were less able to form biofilm than dispersed cells, suggesting that for this system, aggregate formation is not a precursor to biofilm formation^5,7,23^.

Biofilm staining assays should in general be treated with caution since, despite our cautious protocol, the necessary washing steps could remove loosely attached cells. With this caveat, our results might suggest that biofilm formation is triggered not by the release of eDNA per se, but rather by another factor that is released upon antibiotic-mediated cell lysis. An alternative hypothesis might be that released eDNA does trigger biofilm formation, but the amount of eDNA in our system may be sequestered by the aggregated cells, such that it is not available to facilitate attachment to the surface. In this scenario, the dispersal of aggregates by DNase treatment or vortexing might release some eDNA, such that it can better facilitate bacterial surface attachment. Time-lapse microscopy of the dynamics of biofilm formation in the presence and absence of aggregates would be an interesting direction for future study.

## Discussion

Microbial aggregation is ubiquitous and has significant physiological, biophysical, and ecological implications, but the factors that trigger aggregation remain poorly understood. In this work, we established and characterized a simple and manipulable model system in which low-dose exposure to *β*-lactam antibiotics causes *E. coli* cells to aggregate in a well-shaken liquid suspension. In this system, aggregation is mediated by the release of DNA when a small subpopulation of cells lyses in response to the antibiotic. Quantitatively, we found that approximately 6.8% of cell lifetimes ended in lysis. While eDNA is essential for aggregation and appears to be a key structural component of the aggregates, other factors may also be required, since purified genomic DNA did not cause aggregation in our experiments. The aggregation did not increase resistance to (other) antibiotics – i.e. the minimal inhibitory concentration – although we cannot rule out that it might alter antibiotic tolerance, as previously reported^2–4,22^. Low-dose mecillinam treatment did promote biofilm formation, consistent with previous work^13–15^, but aggregation per se did not. Taken together, our results suggest a picture in which lysis of a few cells mediated by low-dose *β*-lactam treatment leads to the release of eDNA which causes aggregation, although other factors may also be involved. This is a relevant finding since suspended aggregates are widespread in the environment and in industry and are increasingly being implicated in disease^2,6,7^.

Although liquid-phase aggregation has not been extensively studied, a growing body of work shows that biofilm formation on surfaces can be triggered by lysis of a subpopulation of cells and the associated release of eDNA^24–26^. This can occur through antibiotic-induced autolysis, via the activation of autolytic enzymes^14,27,28^, via the activation of prophages or upregulation of phage genes, leading to “explosive cell lysis”^13,29^, or through direct lytic action of antibiotics such as *β*-lactams^13,15^ or detergents^15,20^. Our work extends this discussion, by showing that a similar mechanism can also mediate the formation of suspended aggregates in liquid cultures. Our work also connects with previous reports that eDNA is generated in *P. aeruginosa* liquid cultures via lysis of a subpopulation of cells; for *P. aeruginosa* this process has been found to be quorum-sensing dependent as well as dependent on flagella and type IV pili^18^.

Motivated by what is known for biofilm formation, we speculate that eDNA release in our system could occur directly through the action of antibiotics on a few susceptible cells, or could require other factors such as prophages or autolytic enzymes. In either case, it is clear that aggregation depends critically on the presence of a small subpopulation of cells that lyse in response to the low-dose antibiotic. Stochastic bacterial response to high doses of antibiotics has been highlighted in several previous studies^48,49^ and is likely to be clinically relevant since the survival of a few cells can allow the regrowth of the entire population. Our work suggests that stochastic responses to low-dose antibiotics could also have important population-level consequences. Since small populations are more susceptible to stochastic effects than large populations^50,51^, this type of aggregation might occur differently in small bacterial populations compared to large ones.

Low concentrations of antibiotics are found in wastewater, rivers, and lakes, and can also occur during antibiotic therapy, agricultural use of antibiotics, or due to ecological interactions between microbes (e.g. in soil). Low-dose antibiotic exposure has been implicated in many microbiological phenomena, including enrichment of antibiotic-resistant mutants, selection for de novo evolution of resistance^12^, increased genotypic and phenotypic variability, and the triggering of quorum-sensing and virulence^12,13^. It is also well known that low concentrations of antibiotics can trigger biofilm formation on surfaces, not only via eDNA release as discussed above, but also via distinct mechanisms in which antibiotics act as signaling molecules, triggering gene regulatory changes associated with adhesion, metabolic stress, and exopolysaccharide production^13,52^. In some cases, low-dose antibiotic exposure can also prevent biofilm formation^52^. Our work shows that the formation of liquid-phase aggregates should also be considered as a possible consequence of exposure to low doses of cell-wall targeting antibiotics. This adds to the reasons to be concerned about low-level antibiotic exposure and strengthens the case for a more extensive study of antibiotic effects at concentrations far below the MIC.

Recent work in *P. aeruginosa* has highlighted the existence of different types of bacterial aggregates formed under different conditions^35^. In contrast to the dense clumps of cells that have been reported for *P. aeruginosa*^1,3,18,22,35^ and *Staphylococcus aureus*^2^, the aggregates formed in our experiments appear to be loose assemblies of cells held together by a network of eDNA strands. Insights into the diversity of aggregate types may be gained from the large body of soft matter physics work on the aggregation of colloidal particles. It is well known that polymers can cause colloidal particles to aggregate via diverse mechanisms. In particular, polymer-mediated aggregation can occur either via entropic depletion interactions (which have also been observed in bacterial suspensions^4,19,53,54^) or via direct bridging of polymers between particles. Polymer bridging seems the most likely mechanism underlying the aggregation that we observe here; it has also been implicated in aggregation in other bacterial systems^55–58^.

Our system may provide a useful experimental tool to study biophysical aspects of DNA-mediated aggregation in general, since aggregation happens under well-shaken, homogeneous liquid culture conditions and the aggregates are easy to sample and image, allowing the dynamics of aggregation formation to be tracked. We also have a well-defined and controllable trigger for aggregate formation via the addition of low-dose antibiotics and we can quantify the rate of antibiotic-mediated lysis. It would be productive to compare aggregation dynamics in our system with that in other aggregating liquid culture systems (e.g. *P*. aeruginosa, where aggregation is mediated by exopolysaccharides and/or eDNA under different conditions^1,3^).

Previous work has suggested that liquid-phase bacterial aggregates might constitute a first step on the pathway to biofilm formation^5,23^ – however, we found that, although low-dose mecillinam treatment does enhance biofilm formation, this is not due to aggregation per se, since disruption of the aggregates by shaking or by DNase treatment leads to even better biofilm formation. To explain why aggregation seems to actually inhibit biofilm formation in our experiments, we speculate that the eDNA in our aggregates might be coated with bacteria, such that it is not available to mediate adhesion to surfaces. The size of the cloudlike aggregates that we observed is similar to the 42 μm^3^ size that has been reported for *E. coli* genomic DNA in solution^59^, suggesting that perhaps each aggregate corresponds to a single chromosome, to which bacteria have attached. These bacteria-coated chromosomes might not be good building blocks for biofilm formation. However, if the aggregates are broken up by shaking or DNase, the eDNA might become available for larger-scale aggregation.

Since very low concentrations of antibiotics are widespread in the environment, it is tempting to speculate on what might be the ecological consequences of such aggregation. Aggregation could certainly alter the environmental niches that are available to microbes^8^. For example, oxygen availability might become limited inside aggregates. Aggregation could also affect transport within a moving fluid. Aggregates are expected to sink faster than unaggregated cells (a crucial factor in ocean carbon dynamics as well as in wastewater treatment), and would also be transported differently in complex fluid flows such as that found in the gut - as suggested in recent work^8^. Furthermore, aggregates may interact differently with host systems such as immune cells. Interestingly, neutrophil cells can immobilize pathogens using eDNA traps (neutrophil extracellular traps, NETS^60^) – perhaps low-dose antibiotics might aid the immune system by having a similar effect. Aggregation via IgA antibodies has also been shown to prevent *Salmonella* cells from invading the gut epithelium^9^.

Although our study focused on *E. coli*, microbes are usually found in diverse communities. Within a microbiome, one would expect different species to show different aggregation responses to different stimuli – and therefore experience different biophysical or ecological consequences. This raises the interesting possibility (also hinted at in previous work^8,9^) that chemical triggers for aggregation, whether antibiotics or host factors, might provide a way to selectively control microbiome composition.

## Methods

### Bacterial Strains

The *E. coli* strains used in the project are shown in Table 2. RJA002 is a derivative of the wild-type strain MG1655 that contains a chromosomal copy of the yellow fluorescence protein gene under the control of the constitutive *λ*P_*R*_ promoter, together with a chloramphenicol resistance cassette, as previously described^61^. AD37 is a motility-deficient strain with paralyzed flagella. It contains a precise deletion of both the *motA* and *motB* genes, which were deleted simultaneously since they are adjacent in *E. coli* MG1655. This deletion was constructed by plasmid-mediated gene replacement using the plasmid pTOF24 into which an 800 bp fragment was inserted which contained 400 bp immediately upstream and 400 bp immediately downstream of *motAB*^62^. This fragment was generated by recombinant PCR using the following four primers -

1. 5′-GCAACTCGAGCCTTGAACAGTGCCCACAAGCAG-3′
2. 5′-GCAAGTCGACGCCTTTCGCTTCTAATGCCAGTT-3′
3. 5′-TGGTCAACAGTGGAAGGATGATGTCCAGCGTGAGCATGGATATAAGCGATTTTT-3′
4. 5′-AAAAATCGCTTATATCCATGCTCACGCTGGACATCATCCTTCCACTGTTGACCA-3′

**Table 2:** Strains used in this study

Strain	Parent	Characteristics	Source
MG1655	–	K-12 wild-type	Allen lab, University of Edinburgh
RJA002	MG1655	YFP	Lloyd et al.^61^
AD37	MG1655	$\varDelta$ *motA* $\varDelta$ *motB*	this study

Restriction sites for XhoI and Sal1 were incorporated into the flanking primers (1 and 2, respectively, sequence underlined) to permit insertion into pTOF24. This plasmid was transformed into *E. coli* MG1655 and used as a substrate for gene replacement via the 400 bp homology arms. Following selection for integration of this fragment by homologous recombination and subsequent deletion of the chromosome site, the sequence was confirmed by sequencing across the deletion using primers specific to flanking chromosomal sites.

### Bacterial growth conditions

In the majority of our experiments, cells were grown in MOPS glucose minimal medium (MOPSGlu). This consists of potassium morpholinopropane sulfonate (MOPS) buffer, to which are added essential nutrients^63^, as well as 0.2% *w*/*v* glucose as a carbon source. The MOPSGlu medium was made in-house, as described by Brouwers et al.^64^. The doubling times of strains MG1655, RJA002, and AD37 on MOPSGlu medium at 37 °C are 60, 70, and 60 ± 5 min, respectively (Supplementary Fig. 1).

To obtain the starting cultures for our experiments, a good-sized colony from an LB agar plate was inoculated into 5 ml MOPSGlu medium in a conical flask. The culture was then maintained in the exponential growth regime with aeration in a shaking incubator (200 r.p.m.) by diluting periodically in the same pre-warmed medium for more than 20 generations to reach a steady-state cell culture. To ensure exponential growth was maintained, sub-culturing was performed before the optical density (OD, measured at 420 nm using a Jenway 7205 spectrophotometer) reached 0.4.

### Induction of aggregation via low-dose antibiotics

To induce aggregation, planktonic bacterial cultures were prepared in steady-state, exponential phase growth via repeated serial dilution as described above. When the culture density reached 1.6 × 10^8^ cells/ml (OD 0.2 *@*600 nm), antibiotics were added at doses corresponding to MIC^OD0.2^/8000 (with the MIC having been measured for correspondingly dense cultures on the same medium; see Table 1). The cultures were then incubated with aeration in a shaking incubator (200 r.p.m.) for periods of approximately 5 h in a conical flask (100 ml flask containing less than 20 ml cell culture) or a falcon tube (10 ml tube containing 1–2 ml cell culture).

### MIC measurements

Our measurements of the minimal inhibitory antibiotic concentration (MIC) were performed according to previously published micro-dilution protocols^65^ with some modifications, in that we increased the incubation time in the presence of antibiotic and we detected growth quantitatively via OD measurement, rather than with the naked eye. The detailed MIC measurement protocol is given as Supplementary Information.

### CFU measurements

The number of live cells was measured as a function of time using colony-forming unit assays (CFU), for strain RJA002, during our aggregation experiments (Supplementary Fig. 1). Samples for CFU measurement were taken directly from the batch culture, and the CFU per ml was measured according to the method of Herigstad et al.^66^. CFU measurements were performed for 3 biological replicates (independent experiments), each of which consisted of 8 replicates (technical repeats). Data were expressed as mean ± standard error of the mean.

### Measuring the rate of antibiotic killing

To measure the rate of bacterial killing by low-dose exposure to mecillinam, we used resazurin sodium salt (Invitrogen), which is a weakly fluorescent, nontoxic, cell-permeable dye, that is converted to the highly fluorescent resorufin in the presence of metabolically active cells. For a bacterial population that is growing exponentially with growth rate *λ* and death rate *d*, the number of cells *N*(*t*) increases exponentially in time according to

1 $$N(t)=N(0){e}^{(\lambda -d)t}.$$

We assume that resazurin (at fixed concentration *c*_*z*_) is converted to fluorescent resorufin at a rate that is proportional to the number of live (hence metabolically active) bacteria. Therefore the concentration *c*_*f*_ of resorufin increases in time according to

2 $$\frac{d{c}_{f}}{dt}=\alpha N(t)=\alpha N(0){e}^{(\lambda -d)t}.$$

This implies that *c*_*f*_ increases exponentially according to

3 $${c}_{f}(t)=\frac{\alpha N(0)}{(\lambda -d)}{e}^{(\lambda -d)t}$$

(assuming *c*_*f*_(0) = 0). Therefore a plot of the logarithm of the resorufin fluorescence intensity as a function of time should show a straight line with gradient *m* = *λ* − *d*. For a culture without antibiotics, for which we assume no killing occurs, the equivalent plot has gradient *m*_0_ = *λ*. The antibiotic killing rate *d*, relative to the growth rate *λ*, can be found by comparing the values of *m* in the presence and absence of antibiotic: *d*/*λ* = (*m*_0_ − *m*)/*m*. Low-dose mecillinam (MIC^OD0.2^/8000; see Table 1) was added to planktonic cultures of *E. coli* strain MG1665 grown at 37 °C in MOPSGlu medium, at optical density OD_600_ = 0.2 (~1.6 × 10^8^ cells/ml as described above). The culture was then transferred into a 96-well black/clear flat-bottomed microplate (Falkon), such that the total volume of cell culture per well was 200 μl. Resazurin was added to each well to a final concentration of 0.044 mM. The plate was incubated for 6 h at 37 °C, using double orbital shaking at 600 r.p.m. in a plate reader (CLARIOstar, BMG), covered with a Breathe-Easy sealing membrane (Sigma-Aldrich). Time series of the fluorescence emission of resorufin was recorded using a 530–560/580–600-Exc./Emi. filter at 12-min intervals. The logarithm of fluorescence intensity was plotted as a function of time and the gradient was obtained for early times (in the interval 48–96 min after adding mecillinam). The experiments (Fig. 1d in the SI file) were repeated with five biological replicates (independent experiments), and each biological replicate encompassed three technical repeats. Data are expressed as mean ± standard error of the mean. Using this procedure, the gradients that we obtained for the plots of the logarithm of fluorescence signal vs time were *m*_0_ = 1.46 ± 0.04/h (in the absence of mecillinam) and *m* = 1.36 ± 0.03/h (in the presence of mecillinam). Therefore, we conclude that the killing rate is *m*_0_ − *m* = 0.1 ± 0.07/h, or equivalently, 6.8 ± 1% of bacterial lifetimes (doubling time about 60 min; MOPSGlu medium; 37 °C) end in lysis in antibiotic-mediated killing. This killing rate is too low to be detected from growth curves measured using optical density or colony-forming units (Supplementary Fig. 1).

### Digestion of eDNA and eProtein in pre-formed aggregates

Aggregated samples of *E. coli* strain MG1665 were created by adding low-dose antibiotics to planktonic cultures grown in MOPSGlu medium at 37 °C, and incubating for 4 h (as described above). Samples were then taken from the cell culture flasks. Deoxyribonuclease I (DNase I) solution (Stem Cell Technology, 1 mg/ml), and Proteinase K solution (Fisher, 500 μg/ml) were used at a range of concentrations, for eDNA digestion and eProtein digestion, respectively. The addition of DNase I at 5%*v*/*v* (DNase I solution: 100 μg/ml) followed by incubation for 10 min completely removed bacterial aggregates generated by mecillinam addition (Supplementary Fig. 4), while DNase I at 10%*v*/*v* (DNase I solution: 1 mg/ml) followed by incubation for 10 min removed aggregates generated by aztreonam addition (Supplementary Fig. 5). We only performed eProtein digestion experiments on aggregates that were generated by adding mecillinam. We added Proteinase K solution to the samples, at final concentrations of 10%, 20%, or 50%*v*/*v*, followed by incubation for 15 min, 30 min, or 45 min at 37 °C (all times with all concentrations). None of these proteinase K treatments had any detectable effect on the aggregates, as observed by microscopy (Supplementary Fig. 6).

### Prevention of aggregation by early addition of DNase I

To test whether aggregation was prevented by the addition of DNase I at the start of the experiment, cultures of strain MG1655 were prepared in steady-state exponential growth (see above), at OD_600_ = 0.2. Mecillinam or aztreonam was added at a low dose (MIC^OD0.2^/8000; see Table 1), in addition to DNase I solution at 5%*v*/*v* (100 μg/ml) for the mecillinam experiments, or 10%*v*/*v* (1 mg/ml) for the aztreonam experiments. The cultures were then followed both via microscopy and via spectroscopy (using a plate reader). For microscopy, the cultures were incubated with shaking (37 °C, 200 r.p.m.) for periods of approximately 5 h in a conical flask. Samples were removed at regular intervals and imaged under the microscope to look for aggregates (see below for imaging protocol). For spectroscopy, the cultures were transferred to a polystyrene microplate with clear flat-bottomed wells (Greiner Bio-One), such that the total volume of cell culture per well was 200 μl. The microplate was incubated in a plate reader (Fluostar Optima) at 37 °C, with double orbital shaking at 600 r.p.m., for 5 h. OD_600_ was recorded every 6 min (Supplementary Fig. 7). The experiments were performed with four technical repeats, and data are expressed as mean ± standard error of the mean.

### Testing whether purified *E. coli* genomic DNA promotes aggregation

To test whether the addition of purified genomic DNA promotes aggregation, in the absence of antibiotic treatment, we prepared steady-state, exponential cultures of *E. coli* strains MG1665 (motile) and AD37 (nonmotile) as described above, on MOPSGlu medium at 37 °C. When the cultures reached OD_600_ = 0.2 or 0.4, deoxyribonucleic acid sodium salt from *E*. *coli* strain B-Type VIII (Sigma-Aldrich), was added at 0.25 μg/ml, 1 μg/ml, 5 μg/ml, 10 μg/ml, and 20 μg/ml final concentrations. The cultures were then incubated with aeration in a shaking incubator (200 r.p.m.) for up to 1.5 h at 37 °C. The samples were imaged (see below for imaging protocol) to assess whether or not aggregates had formed. No aggregates were observed under any of the conditions tested (varying initial OD, concentration of added DNA, strain MG1655 vs AD37).

Taking the genome size of *E. coli* to be 4 × 10^6^ base pairs and the average molecular weight per base pair to be 660 Da^67^, 1 μg of the gDNA used in our experiments is equivalent to approximately 2 × 10^8^ genomes. The concentration range of gDNA that we used, 0.25–10 μg/ml, is therefore equivalent to approximately 5 × 10^7^–2 × 10^9^ genomes/ml. This should be compared with the estimated number of genomes that are released by cell lysis in our aggregation experiments with low-dose mecillinam. In those experiments, we determined that 6.8% of cell lifetimes end in lysis. During the experiment the cell density increases from approximately 10^8^ cells/ml to approximately 10^9^ cells/ml (Supplementary Fig. 1), implying 3–4 doublings. The number of genomes/ml released by lysis in 3 doublings can be estimated as 0.068 × 10^8^ = 6.8 × 10^6^ (first doubling), 2 × 6.8 × 10^6^ = 1.36 × 19^7^ (second doubling) and 4 × 6.8 × 10^6^ = 2.72 × 10^7^ (third doubling). The total number of genomes/ml released by antibiotic-mediated lysis is therefore estimated to be approximately 5 × 10^7^ for 3 doublings. For 4 doublings this would be 10^8^. Thus, the amount of purified gDNA added in our experiment (5 × 10^7^–2 × 10^9^ genomes/ml) is similar to the estimated amount of genomic DNA released by antibiotic-mediated lysis in our aggregation experiments (5 × 10^7^–1 × 10^8^ genomes/ml).

### Microscopy

Image acquisition was performed using an inverted epifluorescence microscope (Ti-U, Nikon) with 10×, 40×, 60×, and 100× (1.45 NA phase oil) objectives in combination with a digital camera (CoolSNAPHQ2, Photometrics). Sample preparation followed different protocols depending on the magnification of the objective. To image with the 100× objective, 5 μl of cell culture was sandwiched between a cover glass and a glass microscope slide, and sealed with VALAP (an equal mixture of Vaseline, Lanolin, and Paraffin). To image at lower magnifications, 15–20 μl of cell culture was sandwiched between a glass cover slip and a glass microscope slide, using a 1 × 1 cm^2^ disposable, double-sided adhesive plastic Gene Frame (Thermo Scientific). Once prepared, the chamber was allowed to stand for a few minutes with the cover glass at the bottom, to allow aggregates to settle onto the cover glass. In all cases, multiple images (1392 × 1040 pixels, 16-bit, 1 × 1 binning) were taken for different parts of the chamber.

In this work, we used two DNA stains: TOTO-1 iodide (TOTO-1, Thermo Fisher) and propidium iodide (PI, Thermo Fisher). The final concentration of each dye was 1%*v*/*v*, which corresponds to 100 μM for TOTO-1, and 100 μg/ml for PI. Prior to imaging, a dye solution was added to the cell culture, which was shaken in the incubator as before for 5 min before being sampled for microscopy. The fluorescence emission of TOTO-1 and PI were recorded using the microscope filters ET-EYFP (Chroma, 49003), and ET-DSRed (Chroma, 49005), respectively, and using the same time interval for excitation/emission for the same dye in all samples.

### Spectrophotometry

To detect eDNA via spectrophotometry (Fig. 5), steady-state, exponential cultures were prepared as described above, at OD_600_ = 0.2. The cultures were then transferred to a 96-well black/clear flat-bottomed microplate (Falkon), at a total volume of 200 μl of culture per well. Low-dose antibiotics, PI, TOTO-1, and DNase I were added to each well, to final concentrations of MIC^OD0.2^/8000 for antibiotics and 1%*v*/*v* for the dyes. The plate was incubated for 5 h at 37 °C, using double orbital shaking at 600 r.p.m. in a plate reader (CLARIOstar, BMG), with the lid on. Time series of the fluorescence emission of PI and TOTO-1 were recorded using a 544/590-Exc./Emi. filter and a 500/520-Exc./Emi. filter, respectively, at 10 min intervals. The experiments were performed in three biological replicates (independent experiments), and the data are expressed as mean ± standard error of the mean, while each biological replicate was performed with three technical repeats.

### Biofilm formation assay

We quantified biofilm formation for (i) unaggregated dense cultures, as a control, (ii) aggregated cultures, that had been treated with low-dose mecillinam, (iii) cultures in which aggregates were mechanically disrupted, and (iv) aggregated cultures in which aggregates were chemically disrupted using DNase I (Fig. 6).

This quantification was performed using a modified version of the standard staining protocol for biofilm biomass quantification^68^ (see the detailed description in the Supplementary Information). Four 50 ml falcon tubes were prepared to contain 5 ml of steady-state cell culture at OD_600_ = 0.2 and low-dose mecillinam (MIC^OD0.2^/8000; see Table 1) was added to 3 of them. The falcon tubes were incubated with aeration in a shaking incubator (200 r.p.m.) at 37 °C for 5 h, after which the presence of aggregates in the samples with mecillinam (and absence of aggregates in the sample without mecillinam) was checked using optical microscopy (20× objective).

For mechanical aggregate disruption, one of the aggregated cultures was transferred to a reagent reservoir (VistaLab Technologies) and mixed 50 times with an 8-channel micropipette (Eppendorf Xplorer, with 350 μl tips, taking up 200 μl per channel at speed setting *#* 8). During this procedure, care was taken to mix the entire culture by moving the pipette around the reservoir. For chemical aggregate disruption, DNase I solution at 5%*v*/*v* (100 μg/ml) was added to another of the aggregated cultures and mixed for 30 s (40 Hz; Fisher Scientific mixer). The culture was then transferred to a reagent reservoir and mixed 5 times with an 8-channel electronic pipette, as above. For both mechanical and chemical disruption, the disappearance of aggregates was checked using microscopy (20× objective). After the cultures had been transferred to a microplate (see below), an 8-channel pipette was used to mix (30 times) the contents of each well to ensure all aggregates were removed.

Biofilm formation was quantified using Safranin-O staining. A detailed protocol is given as Supplementary Information.

### Statistical analysis

All statistical analyses were performed by using Matlab R2019a (MathWorks; Natick, MA, USA). One-way ANOVA was applied and the results with *p* < 0.05 were considered statistically significant.

### Reporting summary

Further information on research design is available in the Nature Research Reporting Summary linked to this article.

## Supplementary information

Supplemental text and figures

Reporting summary

## Data Availability

The datasets and original code generated during the current study are available from the corresponding author upon reasonable request.
